# Procedural sedation and analgesia for percutaneous trans-hepatic biliary drainage: Randomized clinical trial for comparison of two different concepts

**DOI:** 10.1515/med-2020-0220

**Published:** 2020-08-28

**Authors:** Alex Zanvettor, Wolfgang Lederer, Bernhard Glodny, Andreas P. Chemelli, Franz J. Wiedermann

**Affiliations:** Department of Anesthesiology and Critical Care Medicine, Medical University of Innsbruck, Anichstrasse 35, 6020, Innsbruck, Austria; Department of Radiology, Medical University of Innsbruck, Innsbruck, Austria; Department of Radiology, Landesklinikum Baden-Moedling, Baden Moedling, Austria

**Keywords:** biliary tract, cholestasis, procedural sedation, analgesia, interventional radiology, remifentanil, piritramide, midazolam, S-ketamine

## Abstract

Procedural sedation and analgesia (PSA) is important during painful dilatation and stenting in patients undergoing percutaneous trans-hepatic biliary drainage (PTBD). A prospective, nonblinded randomized clinical trial was performed comparing different analgesic regimens with regard to the patient’s comfort. Patients were randomly assigned to two treatment groups in a parallel study, receiving either remifentanil or combined midazolam, piritramide, and S-ketamine. The primary study endpoint was pain intensity before, during, and after the intervention using the numerical rating scale (0, no pain; 10, maximum pain). The secondary study endpoint was the satisfaction of the interventional radiologist. Fifty patients underwent PTBD of whom 19 (38.0%) underwent additional stenting. During intervention, the two groups did not differ significantly. After the intervention, the need for auxiliary opioids was higher (12.5% vs 7.7%; *p* = 0.571) and nausea/vomiting was more frequently observed (33.4% vs 3.8%; *p* = 0.007) in patients with remifentanil than in patients with PSA. Overall, 45 patients (90.0%) needed additional administration of non-opioid analgesics during postinterventional observation. Remifentanil and combined midazolam, piritramide, and S-ketamine obtained adequate analgesic effects during PTBD. After the intervention, medications with antiemetics and long-acting analgesics were more frequently administered in patients treated with remifentanil (EudraCT No. 2006-003285-34; institutional funding).

## 1 Introduction

Percutaneous trans-hepatic biliary drainage (PTBD) is a common therapeutic modality in the treatment of biliary obstruction, in particular when surgical repair is not practicable and endoscopic retrograde cholangiopancreatography and stenting have failed [1]. In patients suffering from neoplasms and metastases involving bile ducts and liver tissue, the intervention all too often aims at palliative treatment [2,3]. Local anesthesia is sufficient for most of the steps of the procedure, but dilatation prior to insertion of the drain can be very painful and frequently requires the services of a skilled anesthesiologist with experience in inducing conscious sedation [4,5,6,7]. Inadequate sedation and analgesia can induce anxiety and rapid, shallow breathing. Patients may move and alter the position when they experience pain, in particular during puncture of the visceral peritoneum. Uncontrollable motions of the patient can even render the intervention impossible.

The synergistic effects of sedative and analgesic agents in procedural sedation and analgesia (PSA) allow low-dose administration of analgesics with fewer side effects [8]. PSA induces decreased levels of consciousness; thus, patients are sleepy but can be aroused by voice and touch [7]. Airway protective reflexes, spontaneous breathing, and cardio-circulatory functions are unaffected [7]. There is a smooth transition from deep sedation to general anesthesia. If indicated, pre-interventional fasting allows PSA to be extended to general anesthesia. Several sedative and analgesic agents are suitable including propofol, etomidate, midazolam, ketamine, dexmedetomidine, and various opioids [9]. The ideal drug accomplishes rapid onset, with short-time action, low side effects, and immediate reversibility, because the measures during PTBD may provoke intense pain sensations of very short duration, such as peritoneal transgressions or balloon dilatation procedures. Remifentanil, a potent opioid with rapid onset and short duration of action, has been reported to be safe and effective during PTBD [10,11]. Elimination by nonspecific plasma esterases does not depend on hepatic and renal functions [12]. On one hand, the short plasma half-life makes remifentanil an attractive treatment option for day-care patients [13]. On the other hand, remifentanil increases post-interventional hyperesthesia [14]. Therefore, we aimed to investigate whether remifentanil without additional medication can ensure the patient’s comfort and adequate level of analgesia during the intervention and the postoperative observation. The objectives of this randomized controlled study were to compare two different analgesic regimens in patients undergoing PTBD with regard to patient comfort and satisfaction of the interventional radiologist.

## 2 Materials and methods

### 2.1 Trial design

A prospective, non-blinded, randomized, controlled clinical trial for comparison of two different analgesic regimens was conducted from August 3, 2006, to June 20, 2008. In a parallel study, we compared conventional analgesic and sedative drugs commonly used in radiological interventions in an allocation ratio of 1:1. The study was approved by the local ethics committee (UN 2667_LEK No. 243/2.2.7) and registered (EudraCT No. 2006-003285-34).

### 2.2 Participants

All patients consecutively enrolled in the Department of Radiology, Innsbruck University Hospital, Austria, for planned PTBD and optional biliary stenting were screened for eligibility.

Inclusion criteria were as follows: age >18 years, pre-interventional fasting >6 h, no premedication, elective procedure, American Society of Anesthesiologists (ASA) class 1–3, and written informed consent to procedure and sedation.

Exclusion criteria were as follows: age <18 years, pre-interventional fasting <6 h, chronic analgesic treatment, emergency, ASA class 4 or 5, known allergy against PSA medication, pregnancy, or lactation period, and lack of written informed consent to procedure and sedation. Written informed consent regarding the procedure and randomized mode of sedation was obtained from all patients before intervention.

### 2.3 Interventions

The intervention was performed in a group of four people including a specialist in anesthesiology and a specialist in radiology, both with long-standing experience in working in outsourced radiology intervention rooms. Assistance was provided by one anesthesia nursing and one radiology technician. Interventions were performed during the daytime usually lasting between 1 and 2 h. Repeated follow-up interventions were not included in this evaluation [15]. Analgesic and sedative drugs commonly used in radiological interventions were remifentanil (Ultiva^®^; GlaxoSmithKline Pharma GmbH), midazolam (Midazolam^®^; ERWO Pharma GmbH), piritramide (Dipidolor^®^; Janssen-Cilag Pharma GmbH), and S-ketamine (Ketanest^®^; Pfizer corporation Austria). Further medications included metamizole (Novalgin^®^; Sanofi.Aventis GmbH), diclofenac (Voltaren^®^; Novartis Pharma GmbH), and paracetamol (Mexalen^®^; Ratiopharm GmbH).

#### 2.3.1 Remifentanil treatment group

After a loading dose of remifentanil (0.1 μg/kgBW/min) administered by an infusion pump (Injectomat^®^; Fresenius Agilia GmbH) for 5 min, dose adjustment according to organ function and anticipated pain was performed with a maximum dose of 0.25 μg/kgBW/min. Perfusion was stopped approximately 5 min before the anticipated end of the intervention.

#### 2.3.2 PSA treatment group (midazolam, piritramide, and S-ketamine)

Midazolam (0.02 mg/kgBW) and piritramide (0.15 mg/kgBW) were administered sequentially. S-ketamine (0.2 mg/kgBW) was administered immediately before the dilatation of the stenosis. During the postoperative observation, either piritramide, in case of strong pain, or nonopioid analgesics such as metamizole, diclofenac, or paracetamol were administered.

#### 2.3.3 Radiological intervention

PTBD and biliary stenting followed a standardized protocol using a guidewire technique [2]. Patients were placed in a supine position with skin cleaned and draped at the site of puncture. Local anesthesia was achieved with a subcutaneous injection of 10–15 mL of 2% lignocaine (Xylocaine^®^, Gebro Pharma). Initial puncture of the bile duct using a 22G needle was performed under sonographic guidance and verified by an injection of contrast medium and radiological control. With a guiding wire technique, a 5F catheter was placed. After dilatation of the stenosis, the catheter was either replaced by a 6F or 7F catheter (Dawson-Mueller, Cook) to allow adequate drainage of bile fluid or a self-expandable metallic stent was inserted and dilated with a balloon catheter to keep the bile duct open.

#### 2.3.4 Anesthesiological monitoring

Anesthesiological monitoring consisted of noninvasive blood pressure, peripheral arterial oxygen saturation, respiratory rate measurement by end-tidal capnography, and transthoracic impedance during the intervention continuously recorded with ECG in all patients. Vital signs were recorded at 5-min intervals; sedation levels were continuously monitored by anesthesiologists. Peripheral venous access was established and kept open by crystalloid infusion. Prior to intervention, all patients got either 75 mg diclofenac or, in case of gastric or renal complaints, 1 g metamizole. During the procedure, all patients received oxygen (2–5 L/min) through a face mask. For comparability, pain intensity was recorded at certain intervals, before the intervention, during local anesthesia, during dilatation, immediately after the intervention and 2 h later at the recovery room.

Procedure-related complications and side effects of medications were recorded during and after the intervention. Complication assessment focused on respiratory parameters (respiratory rate and peripheral oxygen saturation), hemodynamic parameters (blood pressure and heart rate), and frequency of postoperative nausea and vomiting (PONV) [16]. Hypotension (systolic BP < 20% baseline), bradycardia (heart rate < 50/min), respiratory depression (respiratory rate < 10, oxygen saturation < 90%), and adverse effects typical of μ-opioids, e.g., nausea, vomiting, pruritus, chest rigidity, and post-interventional shivering in the absence of biliary sepsis, were recorded. Complication management included chin lift or jaw thrust to relieve airway obstruction placement of an oral or nasal airway and positive pressure ventilatory assistance. Although not ideal, specific reversal agents for opioids and benzodiazepines were available to be administered as a last resort. Patients were discharged from the recovery room when fully alert and responsive, with intact gag and cough reflex, proper hydration, and an empty bladder.

### 2.4 Outcomes

The primary study endpoint was pain intensity before, during, and after the intervention.

Pain intensity was recorded as a psychometric response using the numerical rating scale (NRS) with categories 1–3 mild pain, 4–6 moderate pain, and 7 or greater severe pain [17].

Secondary endpoints included complications and radiologist’s satisfaction with PSA. The radiologist’s satisfaction with PSA was categorized as 1 excellent, 2 moderate, and 3 fair.

### 2.5 Sample size

Power analysis revealed >80% significance with a sample size of 50 and anticipated differences in VAS score exceeding 2 ± 2 points.

### 2.6 Randomization

**Figure 1:**
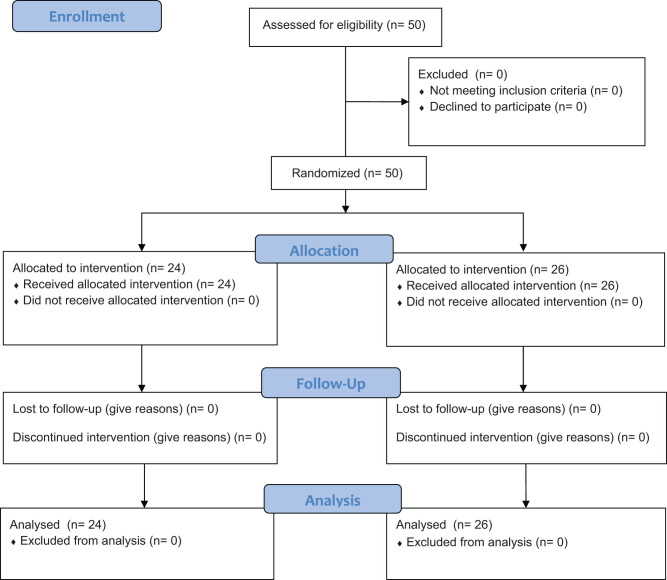
Flowchart of a parallel randomized trial of two groups, modified from the Consolidated Standards of Reporting Trials 2010.

Patients were randomly assigned to two treatment groups by random generators (Figure 1) [18].

The radiologist assigned patients to interventions. The anesthetist generated the random allocation sequence and enrolled the patients the day before the operation. Neither the radiologist nor the investigating anesthetist was blinded to interventions.

### 2.7 Statistical analysis

The null-hypothesis (Ho: μ-Diff = 0) did not expect differences between the two groups, while the alternative hypothesis (H1: μ-Diff <> 0) did. The Kolmogorov–Smirnov test was used for the assessment of normal distribution. Statistical methods used to compare groups for primary and secondary outcomes included variance analysis for repeated measurements were carried out. A *p*-value of <0.05 was deemed significant.

## 3 Results

A total of 50 consecutive patients (29 men, 21 women; mean age 59.9 + 15.1 years; range 27–89 years) scheduled for PTBD were enrolled in this prospective study (Figure 1). Supplementary stenting was performed in 19 (38.0%) cases. Twenty-four patients were randomly assigned to the remifentanil group and 26 patients to the PSA group. Diagnoses included malignant disease (*n* = 42; 84.0%), biliary stone disease (*n* = 3; 6.0%), and benign biliary stricture (*n* = 5; 10.0%). Trans-hepatic punctures were performed under sonographic and fluoroscopic guidance. The majority of patients were ASA grade III (Table 1).

**Table 1:** Demographics, clinical findings, and PONV in 24 patients treated with remifentanil (group 1) and in 26 patients treated with combined midazolam, piritramide, and S-ketamine (group 2)

Characteristics	Group 1 (*n* = 24)	Group 2 (*n* = 26)	*P*
Demographics			
Age (years; range)	61.2; 34–84	58.7; 27–89	0.555
Height (cm; range)	173.2; 149–188	170.6; 149–191	0.392
Body weight (kg; range)	74.0; 40–115	67.9; 35–115	0.205
Body mass index (kg/m^2^; range)	24.5; 17.8–33.2	22.9; 14.0–33.2	0.153
Male (*n*; %)	15; 62.5%	13; 50.0%	
Clinical findings			
Mean systolic blood pressure (mmHg)	150.0	142.0	0.736
Mean diastolic blood pressure (mmHg)	84.6	83.0	0.539
Mean heart rate (n/min)	85.9	77.4	0.037
Mean respiratory rate (*n*/min)	11.0	16.0	0.479
Mean peripheral oxygen saturation (%)	97.1	98.1	0.035
PONV (*n*; %)	8; 33.4	1; 3.8	0.007

### 3.1 Pain intensity indicated by NRS scores

Mild pain was experienced by 25 patients (50.0%) while 17 patients (34.0%) experienced moderate pain during the intervention (Table 2). Postoperative NRS scores did not differ significantly, but severe pain was observed in two patients (8.3%) of the remifentanil group necessitating immediate administration of piritramide. In patients with remifentanil, the need for auxiliary opioids was higher (12.5% vs 7.7%; *p* = 0.571) than in patients with PSA. Overall, 45 patients (90.0%) needed additional administration of non-opioid analgesics such as metamizole, diclofenac, or paracetamol during postoperative observation (Table 2).

**Table 2:** Pain intensity and radiologist’s satisfaction with PSA in 26 patients treated with remifentanil (group 1) and 24 patients treated with midazolam, piritramide, and S-ketamine (group 2)

Characteristics	Group 1 (*n* = 24)	Group 2 (*n* = 26)	*P*
Pain intensity: NRS; 0–10			
Before intervention	0.75	0.42	0.374
Local anesthesia	2.08	2.35	0.291
Dilatation	2.38	3.19	0.224
After intervention	1.21	0.42	0.131
After 2 h observation	0.79	0.46	0.200
Radiologist’s satisfaction with PSA: NRS; 1–3			
During intervention	1.08	1.0	0.293

### 3.2 Complication management

There were no major procedure-related complications. Bradycardia was observed in ten patients (20.0%). Marked hypotension and significant respiratory depression did not occur. None of the patients experienced postoperative shivering. PONV during or immediately after the procedure was more common in the remifentanil group (33.4% vs 3.8%; *p* = 0.007) than in patients with PSA (Table 1). This was treated by administration of 4 mg ondansetron. No itching or muscle rigidity after administration of analgesic and sedative medication was observed. Antagonists for opioids and benzodiazepine were not indicated in any patient.

### 3.3 Radiologist’s satisfaction with remifentanil and PSA

The radiologist reported excellent satisfaction with both anesthesia regimens – remifentanil and PSA – in 49 of 50 patients (Table 2). In one patient of the remifentanil group, only moderate satisfaction was expressed by the radiologist. Irregular breathing under remifentanil treatment aggravated puncture of the bile duct. Even worse, when the dosage was reduced, the patient became agitated.

## 4 Discussion

Both regimens provided adequate analgesic effects during the intervention. Adequate postprocedure analgesia contributes substantially to patient’s comfort, and parenteral opioids remain the mainstay of pharmacologic treatment of acute pain. As maximum pain during PTBD is expected at the end of the procedure, strong analgesics with a short half-life are the preferred drugs. This favors remifentanil, a potent analgesic characterized by rapid onset and offset with a terminal half-life of 10–20 min. Context-sensitive half-life time is approximately 4 min after the infusion is stopped [6,7]. Remifentanil has been reported to be a suitable option for analgesia during painful interventional radiology procedures, in particular during PTBD [11]. However, despite an increased risk in depressed respiration, long-acting opioids are preferable if prolonged pain is expected during postoperative observation. In our study, the frequent need for opioid drugs in the remifentanil group diminished the benefits of the short-time properties of this drug [13].

It is difficult to predict the likelihood of developing nausea and vomiting in patients suffering from bile duct diseases. Furthermore, bag-mask ventilation in patients with bile duct diseases may carry a certain risk of vomiting and aspiration. In our study, PONV was observed more frequently with remifentanil, when administered during general anesthesia. PONV from remifentanil when administered during general anesthesia is not frequently observed [19]. However, the findings of our study indicate that PONV from remifentanil seems to be more frequent under sedation analgesia, underlining the necessity of anti-emetic prophylaxis before analgesia with remifentanil.

Furthermore, in patients who are not intubated, remifentanil produces respiratory depression in a dose-dependent manner, and accidental overdose of the drug can cause severe respiratory depression and respiratory arrest [20]. As titration of opioids is limited by specific ceiling effects, sudden intense pain is best controlled with ketamine as rescue analgesia. However, the radiologists should always give notice of anticipated painful episodes during the procedure. The option of rescue analgesia is not a substitute for lack of interdisciplinary communication.

Administration of antagonists for opioids and benzodiazepine was not needed during our study. Anyway, the reversal of severe respiratory depression and muscle rigidity from opioids by the specific antidote is not ideal as this can lead to acute pain and sympathetic hyperactivity requiring intense pain control thereafter. Another disadvantage of remifentanil is depressed respiratory response to hypoxia with the insufficient stimulus to trigger breathing [20] – altered breathing behavior can impair the progress of the PTBD itself as well. While oxygenation is supported by supplemental oxygen, ventilation can be impaired due to a declined respiratory rate and diminished chest expansion. Capnography using nasal cannulas or face masks with a sampling port for end-tidal CO_2_ monitoring provides noninvasive monitoring of ventilation rate and can enable early recognition of altered respiration patterns. Beyond that, assessment of accurate CO_2_ levels could help to detect hypercarbic patients before the presentation of hypertension, tachycardia, dysrhythmias, and acidosis.

The desirable characteristics of PSA medications include rapid onset of action, short duration of drug effect, rapid recovery time, reversibility with the antidote, reduction of patient anxiety, preservation of patient abilities, and few adverse effects on cardiovascular and respiratory functions. Multimodal analgesia defines the combined administration of opioids and nonopioids to take full advantage of the analgesic effects of each component while minimizing potential side effects. Combined administration of sedating, analgesic, and dissociative medications as used in this study, namely midazolam, piritramide, and S-ketamine, provided adequate analgesia. We are aware that accurate assessment of pain intensity using the NRS is less reliable in sedated patients. Furthermore, interventions might have been more painful in 19 patients who underwent additional stenting.

Complications were rarely observed during our study. Marked hypotension related to myocardial depression or preexisting myocardial dysfunction or hypovolemia and blunting of the sympathetic nervous system response was not observed. Inadequate sedation and analgesia were observed in one patient in the remifentanil group during the intervention. The agitation and uncontrollable motion of the patient during the intervention are disagreeable and may even cause the failure of the procedure. Remifentanil is highly cost-effective and comparable to PSA, but when patients need overnight observation in the hospital, costs multiply very quickly.

In conclusion, both regimens, remifentanil and PSA with combined midazolam, piritramide, and S-ketamine proved to be effective in providing adequate analgesic effects during PTBD. Patients treated with remifentanil more frequently needed antiemetic medication and additional long-acting analgesic medications after the intervention.
